# Mad honey (wild honey) poisoning: clinical case series from Nepal

**DOI:** 10.1097/MS9.0000000000002448

**Published:** 2024-08-14

**Authors:** Anil Jung Thapa, Subodh Chapagain, Samit Lamichhane, Egesh Aryal, Aashish Sapkota, Abishkar Ghimire, Bikash Bhatt, Sweta Agarwal, Aayushma Khadka, Suraj Parajuli

**Affiliations:** a Nepal Army Institute of Health Sciences; b Metro Kathmandu Hospital, Maharajgunj, Kathmandu; c Manosamajik Apanga Bandi Hospital, Lalitpur; d Chitwan Medical College, Bharatpur, Nepal

**Keywords:** grayanotoxin poisoning, Himalayas, mad honey

## Abstract

**Introduction:**

Mad honey is commonly used for hypertension, and coronary artery disease, and as a sexual stimulant. Patients with mad honey poisoning present with dizziness, nausea, syncope, blurred vision, bradycardia, and hypotension with ECG findings of sinus bradycardia, complete AV block, and ST elevation.

**Case discussion:**

Here, the authors report five cases admitted to our tertiary care center following the consumption of mad honey. The amount of ingestion of honey varies from 1 to 2 teaspoons (~10–20 ml). Most of the cases presented with chief complaints of nausea, dizziness, and vomiting, and all the cases had hypotension and bradycardia. Two cases were admitted to the ward and three of them were admitted to the ICU for further management. They were managed with intravenous fluid, injection atropine along with adjunctive vasopressor and oxygen whenever necessary.

**Discussion:**

Mad honey contains grayanotoxin extracted from the nectar of Rhododendron species. This honey contains grayanotoxin, which binds to sodium channels in its open state causing hyperpolarization of the sodium channel predominantly causing gastrointestinal, neurological, and respiratory symptoms. Intravenous fluids and injection atropine are the mainstays of management in an ICU setup. Some also may require vasopressors.

**Conclusion:**

Mad honey poisoning is rare, and limited cases have been reported in Nepal. Physicians should consider mad honey poisoning in cases with ingestion history and clinical symptoms, as it may be a clinical diagnosis due to limited lab tests for grayanotoxin intoxication. Supportive management still forms the cornerstone for its management after diagnosis.

## Introduction

Highlights

- Mad honey is commonly used for hypertension, and coronary artery disease, and as a sexual stimulant.
- Patients with mad honey poisoning present with dizziness, nausea, syncope, blurred vision, bradycardia, and hypotension with ECG findings of sinus bradycardia, complete AV block, and ST elevation.
- Intravenous fluids and injection atropine are the mainstays of management with or without vasopressors.

Mad honey is extracted from the nectar of Rhododendron species (*Rhododendron ponticum* and *Rhododendron luteum*), which are found in the upper belt of the Himalayans in Nepal. They are also found in the mountains of the eastern black sea region of Turkey and some parts of North America, Brazil, and Europe^1^. This honey contains grayanotoxin, which binds to sodium channels in its open state causing hyperpolarization of the sodium channel predominantly causing gastrointestinal, neurological, and respiratory symptoms^2–4^. Patients with mad honey poisoning present with dizziness, nausea, syncope, blurred vision, bradycardia, and hypotension with ECG findings of sinus bradycardia, complete AV block, and ST elevation^5^. No routine tests are available to measure grayanotoxin. Thus, the alleged history of consumption of honey along with the above-mentioned signs and symptoms excluding other diagnoses are enough for the diagnosis of poisoning. Most cases require ICU admission and are managed with intravenous fluids and atropine^6^. Here, we described a case series of five patients who presented to a tertiary health care center with mad honey poisoning who had been admitted and managed in ward and ICU. It has been reported in line with PROCESS 2020 guidelines^7^.

## Case discussion

### Case 1

A 29- year-old male, presented to ER on 22 February 2023, with complaints of vomiting for multiple episodes and dizziness for 2 h with an alleged history of ingestion of one tablespoon of honey. The patient had no history of hypertension, diabetes mellitus, or any other underlying diseases. At presentation, he was ill-looking and drowsy with a blood pressure of 80/40 mmHg and a heart rate of 48 bpm. At emergency, he was managed with intravenous fluids, atropine 2 mg, and noradrenaline infusion and was admitted to the intensive care unit for further management and observation. On investigation, he had a serum creatinine level of 1.6 gm/dl with other parameters within normal limits. During the ICU stay, he had multiple episodes of desaturation and was kept on 1 l/min of oxygen. He was kept under observation for one day and as his vitals were stable, he was shifted to the ward for further observation and was discharged after hemodynamic stability.

### Case 2

A 45-year-old male, presented to ER on 31 October 2022, with the complaint of multiple episodes of vomiting following the consumption of about one teaspoon of wild honey. The patient had no history of hypertension, diabetes mellitus, or any other underlying diseases. During the presentation, he was ill-looking and was hypotensive with a blood pressure of 60/40 mm of Hg, had bradycardia with a pulse rate of 45 beats per min, a respiratory rate of 17 breaths per minute, 97 F of body temperature and oxygen saturation of 94% in RA. His lab parameters were within normal limits. He was primarily managed in the emergency room with IV fluids, injection of adrenaline, hydrocortisone, pheniramine maleate, pantoprazole, and ondansetron. The patient was then shifted to the ward for conservative management and observation. He was discharged the following day with hemodynamical stability.

### Case 3

A 51-year-old male presented to ER on 31 October 2022, with the complaint of multiple episodes of vomiting after the consumption of about one spoon of wild honey. The patient had no history of hypertension, diabetes mellitus, or any other underlying diseases. During the presentation, he was ill-looking with a blood pressure of 100/60 mm hg, heart rate of 48 beats per min, respiratory rate of 16 breaths per min, temperature of 98F, and oxygen saturation of 94% in room air. Lab parameters were normal. He was managed in the casualty department with IV fluids, injection of hydrocortisone, adrenaline, pheniramine maleate, pantoprazole, and ondansetron. For observation and conservative management, the patient was shifted to the ward and was discharged the following day with hemodynamical stability.

### Case 4

A 46-year-old male presented to ER on 31 October 2022, with symptoms of shortness of breath and multiple episodes of vomiting following the consumption of wild honey (about 2 tablespoons). The patient had no history of hypertension, diabetes mellitus, or any other underlying diseases. During presentation, the patient was hypotensive with a blood pressure of 90/60 mm of Hg, a pulse of 46 beats per min, a respiratory rate of 14 breaths per min, 98.6F body temperature, and 96% of SpO2 in room air. His laboratory findings were 140 mEq/l sodium, 3.7 mEq/l potassium, 25 mg/dl urea, 1.3 mg/dl creatinine, 16.3 g/dl hemoglobin, TLC 9500/ ul, platelets 236 000/ ul, 0.59 mg/dl total bilirubin, 0.23 mg/dl direct bilirubin, 36 U/l AST, 41 U/l ALT, 104U/l ALP, Urine routine examination normal, PT/INR 11 and 0.9 respectively. He was admitted to ICU and managed with atropine, hydrocortisone, adrenaline, ondansetron, anti-histaminic, and IV fluids, all in injectable forms. After hemodynamical stability he was shifted to the ward and was discharged the following day.

### Case 5

A 51-year-old female patient was admitted to our service on 16 April 2023, with symptoms of multiple episodes of vomiting and facial swelling after consumption of wild honey. The patient had no history of hypertension, diabetes mellitus, or any other underlying diseases. At presentation, she was hypotensive with a blood pressure of 70/50 mm of Hg, had bradycardia with a pulse of 47 beats per min, respiratory rate of 16 breaths per min, and 94% of Spo2 in room air. Her laboratory findings were 13.5 g/dl Hb, 8300/ ul TLC, 30 mg/dl urea, 0.8 mg/dl creatinine, 144 mEq/l sodium, 4.2 mEq/l potassium, urine routine examination normal, 0.99 mg/dl total bilirubin, 0.39 mg/dl direct bilirubin, 21 U/l AST, ALT 30 U/l, 119 mg/dl of blood sugar. She was managed with injectable atropine, hydrocortisone, noradrenaline, anti-histaminic, and fluids in the emergency department. After that, she was admitted to ICU and managed with atropine, ondansetron, adrenaline infusion, enoxaparin sodium, and oxygen supplementation. After hemodynamical stability he was shifted to the ward and was discharged the following day.

All the cases are summarized in Table 1.

**Table 1:** Summary of all cases

	Case 1	Case 2	Case 3	Case 4	Case 5
Age	29 years	45 years	51 years	46 years	51 years
Sex	Male	Male	Male	Male	Female
Amount of ingestion (teaspoon)	One (10 ml)	One	One	Two	One
Symptoms	Vomiting and dizziness	Vomiting	Vomiting	Vomiting and shortness of breath	Vomiting and facial swelling.
Blood pressure (mm of Hg)	80/40	60/40	100/60	90/60	70/50
Heart rate (beats per min)	48	45	48	46	47
Serum creatinine (mg/dl)	1.6	0.9	1	1.3	0.8
Signs of anaphylaxis	No	No	No	Yes	Yes
ICU admission	Yes	No	No	Yes	Yes
Medication: atropine (mg)	2	—	—	2	2
Noradrenaline infusion	Yes	—	—	Yes	Yes
Oxygen requirement	Yes	No	No	Yes	Yes
ICU admission (h)	24	—	—	24	24
Admission time (h)	48	24	24	48	48
Follow-up/complications	None	None	None	None	None

## Discussion

Mad honey is a natural product made by bees in the Himalayan region from Nepal, the Black Sea region of Turkey, and various parts of North America, Brazil, and Europe. It contains grayanotoxin extracted from the nectar of Rhododendron species (*Rhododendron ponticum* and *Rhododendron luteum*)^1^. Honey is regarded as a herbal medicine in this part of the world. So, people tend to consume it consciously rather than accidentally^4^.

The earliest reports of mass mad honey poisoning were explained by Xenophon (a military commander) back in 401 BC in his book Anabasis^8^. Mad honey is used as an aphrodisiac (sexual stimulant), and alternative therapy for gastrointestinal disorders like peptic ulcer disease, gastritis, dyspepsia, bowel disorders, arthritis, skin ailments, colds, and hypertension. The consumption of about 15–30 gm of mad honey leads to intoxication, and symptoms start to appear after half to four hours^6^.

Like other previous case series male population is found to be presented in the hospital with poisoning following ingestion of mad honey^9–11^. This can be explained by the male tendency to use mad honey for improving sexual performance^6^.

More than 20 different types of grayanotoxin have been found among which GTX1 AND GTX 2 are frequently found in mad honey. A case report from Portugal reported a higher amount of GTX1 in Nepalese mad honey causing bradycardia and hypotension despite a small dose. In six cases reported from Korea, the minimum concentration of GTX1 causing symptoms is between 2.52 and 4.55 ng/ml^12^.

The amount of intake and symptoms correlate variably as the concentration of grayanotoxin is not uniform in locally available products. Various study reports the varying amount of ingestion for the development of symptoms, as Yilmaz reported (5–30 g). In our case series, the amount of ingestion was similar to the amount taken as reported by Baniya and colleagues and Paudel and colleagues, and case reports across Nepal report a minimum of 1.5–2 teaspoons^11,13,14^. A meta-analysis of 1199 cases of mad honey poisoning reports 1–5-tablespoon ingestion of mad honey by 75.43% of cases reviewed in that study^5^. The amount of intake and presentation of symptoms is believed to have seasonal variation, which is yet to be studied. Since most of the studies are retrospective, no studies explain the variation in the amount of honey ingestion along with the change in seasons.

Mad honey contains grayanotoxin, which acts by binding to voltage-gated sodium channels in its open state causing persistent depolarization of sodium channels in muscles and neurons. This produces the effects of bradycardia, hypotension, and decreased respiratory effects^2^. The action of grayanotoxin present in honey affects the central nervous system causing respiratory depression and bradycardia, which has been described by Onat and colleagues in a rat study where intracerebral and intraperitoneal injection of the toxin was done and compared. This study also describes vagus-mediated bradycardia following ingestion of grayanotoxin-containing mad honey^15^. The cardiac effects of grayanotoxin have been best described by its M_2_ receptor agonist effects since the symptoms of bradycardia are reversible by AF-DX 116^3^. Rat study of renal and hepatic effects of grayanotoxin I showed that on high doses can cause hematuria, proteinuria, and increased liver enzymes^16^. However, no literature reports about increased serum creatinine levels following ingestion of mad honey as seen in our case series. This is probably due to hypotension leading to acute kidney injury (AKI). Grayanotoxin poisoning can be put under cholinergic toxidrome as it contains most of the features and can be reversed by atropine^17^.

The onset of symptoms following ingestion of mad honey ranges from 30 min to 4 h as reported by previous studies. In a case series of 21 patients, Demir *et al.* ^9^ found the mean onset of symptoms to be 3.4 h. However, Sohn *et al.* ^10^ report the mean time for the onset of symptoms following ingestion of Nepalese honey to be 34 min.

More than 40 different symptoms following grayanotoxin poisoning have been described in studies^5^. Commonly encountered symptoms and signs are dizziness, weakness, syncope, nausea, vomiting, hypotension, and bradycardia^6,9^. Some of the uncommon symptoms are A-V block^18^, Atrial fibrillation^19^, and asystole^20^. Most of the bradycardia cases respond to IV fluids and Atropine as happened in our cases. Rarely, a pacemaker may be required to manage bradycardia as reported by studies^5^.

Diagnosis of mad honey poisoning is based on high clinical suspicion, as the investigation has very limited values. Some of the investigation available is liquid chromatography with mass spectrometry method, which has limited clinical values and is used only for research purposes. All the cases following grayanotoxin poisoning are advised to be admitted to the ICU for further management. Intravenous fluids and injection atropine are the mainstay of management^6^. Three of our cases required vasopressor for the management of hypotension, which is similar to Setareh-Shenas *et al.* ^21^ and Baniya *et al.* ^13^. Most of the cases recover within 24–48 h of ingestion of mad honey^5,9,10^, which is similar to our case series. There has been no mortality reported till now from the consumption of mad honey^5^. This finding is consistent with our case series.

## Conclusion

Mad honey poisoning is rare, and limited cases have been reported in Nepal. Physicians should suspect mad honey poisoning in a case with a history of ingestion and clinical presentation as it could be a clinical diagnosis alone due to the lack of availability of lab measures to prove grayanotoxin intoxication. Supportive management still forms the cornerstone for its management after diagnosis.

## Ethical approval

There is no need for ethical approval for case series according to the local ethical guidelines.

## Consent

Written informed consent was taken from individual patients.

## Source of funding

Not applicable.

## Author contribution

A.J.T.: supervision, writing, editing, submission. S.C.: writing, editing. S.L.: writing, editing. E.A.: writing, editing. A.S.: writing, review. A.G.: writing, review. B.B.: writing, review. S.A.: writing, editing. A.K.: writing, editing. S.P.: writing, editing, supervision.

## Conflicts of interest disclosure

The authors declare no conflicts of interest.

## Research registration unique identifying number (UIN)

Not applicable.

## Guarantor

Anil Jung Thapa.

## Data availability statement

The complete data of this research are fully available on request made to the corresponding author.

## Provenance and peer review

Not applicable.
